# Organizational Practices and Their Outcomes for Employees with Disabilities: A Review and Synthesis of Quantitative Studies

**DOI:** 10.1007/s10926-025-10283-6

**Published:** 2025-03-12

**Authors:** Rik van Berkel, Eric Breit

**Affiliations:** 1 Utrecht School of Governance, Utrecht University, Bijlhouwerstraat 6, 3511ZC Utrecht, Netherlands; 2 Department of Leadership and Organizational Behaviour, BI Norwegian Business School, Nydalsveien 37, 0484 Oslo, Norway

**Keywords:** Performance, Well-being, Sustainable employment, People with disabilities, Organizational practices, Review

## Abstract

**Purpose:**

This review and synthesis aims to answer the following question: what do existing empirical studies tell us about the relationship between organizational practices and their outcomes in terms of the performance, well-being and sustainable employment of employees with disabilities?

**Methods:**

This review builds on a scoping review of empirical studies of organizational practices aimed at the hiring and retention of people with disabilities. It focuses on a specific group of studies identified in the scoping review: studies examining outcomes of organizational practices for employees with disabilities (EWD). Additional selection criteria were: (1) studies focused on the performance, well-being and sustainable employment of EWD; (2) studies used quantitative methods; and (3) studies were published in high-quality journals. This resulted in 27 articles.

**Results:**

Three clusters of organizational practices received most attention in the articles: workplace relations and organizational culture; accommodations; and leadership. The studies found significant positive and negative relationships between practices in these clusters and the three outcomes mentioned above. These empirical findings were supported by the theoretical perspectives used in the studies. Although diverse, most of these theoretical perspectives share their focus on interactions between individuals and their (work) environment.

**Discussion:**

We recommend that future research into the outcomes of organizational practices for EWD should be both broader (examining more clusters of practices and their outcomes for people with and without disabilities) and deeper (examining similar practices-outcome combinations in different contexts). This will increase our understanding of what practices work for whom under what circumstances.

**Supplementary Information:**

The online version contains supplementary material available at 10.1007/s10926-025-10283-6.

## Introduction

This article presents the results of a review and synthesis of quantitative studies of the outcomes of organizational practices for employees with disabilities (EWD). The Preamble to the United Nations Convention on the Rights of Persons with Disabilities [1] defined disability as the result of the interaction between persons with impairments and attitudinal and environmental barriers. This concept of disability is reflected in demand-side oriented studies that examine how employers facilitate or hinder the labor-market participation and successful performance and participation of people with disabilities [2–8]. A significant proportion of these studies examined recruitment and selection processes [9–11]. However, the successful performance and participation of EWD *in* organizational contexts needs attention as well [6, 12–15], and research should address organizational practices across the employment cycle [16].

This article focuses on organizational practices and outcomes for EWD, i.e., people with disabilities in employment. We focus on three groups of outcomes: performance, well-being, and sustainability of employment. These outcomes are the ones most frequently addressed in studies of organizational practices and their outcomes, and not only relevant for EWD but also for the organizations where they work. The general research question is: what do existing empirical studies tell us about the relationship between organizational practices and their outcomes in terms of the performance, well-being, and sustainable employment of EWD? We answer this question in three steps. First, we cluster the organizational practices that the articles in our review analyzed. This step is necessary because bundling similar practices in clusters helps to reduce the variety of the organizational practices that have been examined. The second step synthesizes the results of the studies: what relationships between organizational practices and outcomes were found? In the third step, we examine the theoretical perspectives that the studies used.

We contribute to existing studies of organizational practices and their outcomes for EWD as follows. In general, we provide an update to already existing reviews of these practices [4, 17–20]. More importantly, our review is—to our knowledge—the first to systematically focus on synthesizing the employee outcomes of a broad range of organizational practices into more meaningful subsets that can be aligned with organizational decision making. Some existing reviews have applied a narrative approach [20, 21] and identified promising employer practices such as accommodations, support and workplace culture. Other reviews focused on specific practices such as customized training [22] or accommodations [23], and showed that there is some evidence that these practices have a positive impact on EWD’s performance. In addition, we contribute by analyzing the theoretical perspectives used in the studies. Combining empirical findings with theoretical perspectives that substantiate these findings strengthens our knowledge base of what works for the successful performance and participation of EWD. Finally, we contribute to attempts to strengthen the links between rehabilitation and human resource management (HRM) research. Various review studies have already pointed at these links [4, 17, 19, 21]. We do so by reflecting on how the organizational practices that according to the studies discussed in our review have positive outcomes for EWD relate to mainstream human resource (HR) practices as identified in HRM studies. This may help HRM researchers to reflect on the issue of whether mainstream HR practices are really mainstream for *all* employees.

## Theoretical Background

### Organizational Practices and HR Practices

Following Stone and Colella’s model [6], organizational policies and practices are an important factor affecting the treatment of people with disabilities in organizations. In their article, these policies and practices are elaborated in terms of human resource policies and practices. In this review, we use the organizational practices terminology for the following reasons.

First, we focus on practices rather than policies. Following the HR value chain logic that explains how HR strategies produce organizational outcomes [24, 25], actual HR practices are the enactment of HR policies and intended HR practices. HR scholars have argued that these actual practices and how employees perceive them are most important in the process linking HR strategies to outcomes [26]. Furthermore, in many organizations, especially small enterprises, formal HR policies may be absent and organizational practices develop in a more informal and ad hoc way. Therefore, focusing on organizational *practices* rather than policies seems appropriate.

In addition, the concept of *organizational* practices is broader than the concepts of HR (or employer [22, 27]) practices. Studies of EWD have shown that their relationships and interactions with colleagues, team members, and supervisors are of importance for EWD’s outcomes [28], but these relationships and interactions are not necessarily subject to HR policies and therefore enactments of these policies. At the same time, these relationships and interactions *could* be made subject to HR policies [22], for example, by facilitating or encouraging colleagues or supervisors to support EWD. Therefore, focusing on *organizational* practices directs attention to practices that may be highly impactful for EWD but are outside the scope of formal HR policies.

### Mainstream HR Practices and Disability-Specific Practices

In their review of HR practices for inclusion, Schloemer-Jarvis and colleagues used the high-performance work practices (HPWP) theoretical framework [19]. Based on Appelbaum and colleagues’ AMO model [29], these HPWP are expected to have positive impacts on employees’ Abilities, Opportunities, and Motivation, which will produce positive employee and organizational outcomes. HR scholars in the HPWP research tradition identified several mainstream HR practices [25, 30, 31]. Boselie distinguished five HPWP: selective recruitment and selection; compensation and performance-related pay; appraisal and performance management; training and development; and employee involvement [25].

Although this list of HPWP could provide us with a way of clustering organizational practices studied in the articles in our review, we decided not to use it. Instead, we clustered the practices inductively and took the organizational practices in the articles rather than a theoretical framework as our starting point. This allows us to compare the mainstream HR practices (the HPWP mentioned above) with the clusters of organizational practices addressed in studies specifically focusing on EWD. This comparison may be relevant against the background of debates on the usefulness of disability-specific organizational practices and of identity-conscious versus identity-blind approaches [19, 32, 33]. When the clusters we find fully coincide with the HPWP mentioned before, and when their outcomes for EWD are positive, investing in HPWP will also be beneficial for EWD. When they do not fully coincide—and that is what we expect—it could indicate that EWD have needs different from those of employees without disabilities, that ask for disability-specific organizational practices. However, it could also mean that HPWP fail to meet needs that are not specific to EWD but are also relevant for other groups of employees. The increasing diversity among employees, of which employees with disabilities are one example, is likely to increase the diversity of employee needs that organizations need to address. In other words, instead of perceiving EWD’s needs as special and requiring attention through disability-specific organizational practices, they may be exemplary for a diversification process of employee needs in general, which might challenge organizations’ perceptions of what are considered as mainstream HR practices. Various studies [32–34] have examined this issue explicitly in the context of accommodations. They found that when accommodations are accessible for employees in general, positive outcomes can be realized for employees with and without disabilities. This brings us to the final issue of this section.

### Disability-Specific Practices, Vulnerable and Non-vulnerable Employees

The positive (enabling) or negative (disabling [35]) effects of organizational practices for EWD tell us nothing about the effects that these practices may have for other groups of employees. Many studies examining outcomes of organizational practices for EWD do not study the outcomes of these practices for other groups of employees, which may unintendedly result in a disability-specific or identity-conscious reading of findings. However, organizational practices that enhance the abilities, motivation, and opportunities of EWD to contribute to the organization where they work, may also be beneficial for other groups of employees. Above we referred to accommodations and that these might benefit all employees. Practices that are considered beneficial for EWD such as support structures and attention for workplace cultures may also be beneficial for other vulnerable groups in organizations such as older workers, religious minorities or LGBT [36–38]. Although our review does not enable us to decide whether the findings point at specific needs of EWD or at needs of EWD that they share with other groups of employees (or a combination of both), raising this issue hopefully contributes to a nuanced interpretation of the findings presented below.

## Methods

### Starting Point: Scoping Review

The basis of this review was a scoping review aimed at mapping the state-of-the-art of empirical research of organizational practices for the hiring and retention of people with disabilities. Elsewhere, we have described the methods used in the scoping review and presented its results [39]. The search string was divided into three groups of search terms: the target group (i.e., people with disabilities); organizational practices; and employment outcomes. In addition, articles were excluded if they were published in a journal without an impact factor. This process resulted in a list of 146 articles. For a schematic overview of the selection process, see Online Resource 1.

The articles were categorized in groups based on content characteristics. One of these groups included 59 studies empirically analyzing relationships between organizational practices and their outcomes for EWD. This group was the starting point of the current review.

### Selecting Articles for This Review

A screening of these 59 articles showed that they varied in terms of the practices and outcomes that were studied. A clustering of the outcomes revealed that three clusters of outcomes were most frequently studied (49 of the 59 articles): performance, well-being, and sustainable employment. Articles studying these outcomes were selected. As a next step in the selection process, we decided to focus on quantitative studies for pragmatic reasons: it would reduce heterogeneity and make a synthesis of findings more feasible, and most of the 49 studies used quantitative methods. Finally, we did a quality assessment of the remaining 30 articles, using a light quality assessment approach. The reasons for this were twofold. First, given the limited number of available studies of outcomes of organizational practices of EWD as well as their heterogeneity, we aimed to give a broad overview of these studies. Secondly, rigorous effect studies such as experiments, RCT’s and longitudinal studies are rare in this area of research [18]. Thus, we followed the approach Schloemer-Jarvis et al. used [19], and retained articles published in journals of the top and second quartiles (Q1 and Q2) in their respective disciplines. For this assessment, we used the SCImago Journal Rank (SJR) indicator, publicly available on the SCImago website (see https://www.scimagojr.com). This assessment resulted in the exclusion of one article. In addition, we removed two articles reporting respondents’ perceptions of effectiveness of organizational practices without any form of testing these perceptions. Of the remaining 27 articles, 17 articles were published in Q1 journals, the others in Q2 journals. Online Resource 2 summarizes the selection process; Online Resource 3 provides an overview of the journal rankings.

### Methodological Rigor of the Studies in the Review

Most studies used surveys as their research method. Some were based on secondary analysis of existing survey or register data of employees with or without disabilities. A limited number of studies used a longitudinal or (semi-)experimental approach. Almost all studies used multiple linear regression, logistic regression or structural equation modeling as their analysis technique. The three oldest studies in our sample [40–42] used analysis of covariance (MANCOVA), correlation or means comparison tests. A few studies [43–45] used different analysis techniques: survival analysis, difference-in-difference estimation and hierarchical linear modeling analysis. With two exceptions all studies used a level of significance of 0.05, considering findings with p-values below 0.5 as significant. The two studies used a significance level of 0.1 [46, 47].

Of course, one may argue that the methodological rigor of the studies differs. For example, structural equation modeling provides more in-depth insight into how independent variables interact in producing certain outcomes than studies based on correlation analyses or regression. And longitudinal studies provide a stronger basis to draw conclusions about causal relationships. Nevertheless, we decided not to attach rigor quality labels to the studies included in this review. Our main reason for this is that our aim was to provide an overview of what we currently know about organizational practices and their outcomes. Even studies with less methodological rigor may be helpful in stimulating further research of practices and their outcomes. In addition, given the heterogeneity of the studies that we discussed above, comparing findings of studies—and using methodological rigor as one of the ways to interpret and value different findings—is hardly feasible.

### Data Extraction and Analysis

The following general information was collected from the articles: author(s), title, year and journal of publication, country of data collection, research objects, practices and outcomes studied, theoretical perspective leading the study’s research question or hypotheses, methods and analysis techniques. The findings of each article were summarized, focusing on findings related to relationships between organizational practices and the three outcomes. In studies that included mediators in their research model, these were included in the summary of results when these mediators represented organizational practices or outcomes.

The organizational practices were clustered inductively, resulting in eight clusters that will be presented in more detail below. The second step synthesized the findings. In their review, Ellenkamp and colleagues concluded that outcomes differed; and that no controlled effect studies have been done for successful interventions [18]. Our review studies revealed a similar picture. Although this makes a meta-analysis of findings impossible, a less robust synthesis can still be useful for researchers and practitioners. By clustering the outcomes and practices, and by summarizing the results of the studies for each of the practice-outcome combinations, we were able to synthesize the findings in a way that fits with the current state-of-the-art in research. In the final step, we mapped the theories the articles used to hypothesize and substantiate relationships between the practices and outcomes their studies addressed.

## Results

### The Articles

The articles were published in 22 different journals (see Table 1). Following the Scopus® Subject Areas and Subject Categories, the journals cover various research subjects (see Online Resource 3): rehabilitation; social sciences health; organizational behavior and human resource management; applied psychology; social psychology; medicine; public health, environmental and occupational health; psychiatry and mental health; and sociology and political science. This illustrates the multidisciplinary character of the research topic. Eleven studies took place in the USA, two in Canada. The remaining studies came from Europe (six), Asia (five), and South America (two). One study took place in three countries (Australia, Canada, and Italy). Using a multi-actor approach is common in this area of research. Most articles used this approach, usually combining employees with and without disabilities or EWD and their supervisors as respondents.

**Table 1:** Article characteristics

Article	Country	Journal	Research objects	Practices cluster	Outcomes cluster	Theoretical perspective
Banks et al. (2001)	USA	Psychiatric Rehabilitation Journal	EWD, vocational professionals	Workplace relations and organizational culture	Performance	
Chandola and Rouxel (2021)	UK	Social Science & Medicine	Employees with and without disabilities	Accommodations	Sustainable employment	ICF model of disability
Chordiya (2020)	USA	Review of Public Personnel Administration	Employees with and without disabilities	Workplace relations and organizational culture	Sustainable employment	Discrimination theory; Optimal distinctiveness theory
Chow et al. (2014)	USA	Psychiatric Services	EWD	Accommodations	Performance; Sustainable employment	ICF model of disability
Coll and Mignonac (2023)	France	International Journal of HRM	EWD, supervisors	Support of EWD	Performance; Well-being	Basic psychological needs theory
De Carvalho-Freitas (2023)	Brazil	Applied Psychology	EWD, supervisors	Accommodations	Performance	
De Carvalho-Freitas and Stathi (2017)	Brazil	Journal of Applied Social Psychology	Students	Workplace relations and organizational culture	Performance	Intergroup contact theory
Eissenstat et al. (2022)	South Korea	Rehabilitation Counseling Bulletin	EWD	Recruitment and selection; Workplace relations and organizational culture; Accommodations	Well-being; Sustainable employment	Theory of work adjustment
Farris and Stancliffe (2001)	USA	Journal of Intellectual and Development Disability	Employers/HR; employees with and without disabilities	Support of EWD	Performance; Sustainable employment	
Flores et al. (2021)	Spain	International Journal of Environmental Research & Public Health	EWD	Practices general	Well-being	JD-R model
Gray et al. (2014)	USA	Work	EWD	Workplace relations and organizational culture; Accommodations; Support of EWD	Well-being	Theory of work adjustment
Kensbock and Boehm (2016)	Israel	International Journal of HRM	EWD	Leadership	Performance; Well-being	Transformational leadership theory
Luu (2018)	Vietnam	Employee Relations	EWD, supervisors	Leadership; Practices general	Well-being	JD-R model; social identity theory
Lyubykh et al. (2020)	USA	Journal of Occupational Rehabilitation	EWD, supervisors	Leadership	Performance; Well-being	Social exchange theory
Man et al. (2020)	China	Frontiers in Psychology	Employees with and without disabilities, supervisors	Accommodations	Performance	Componential model of individual creativity; self-efficacy theory
Mank et al. (2000)	USA	Mental Retardation	Vocational professionals	Support of EWD	Performance; Sustainable employment	
Novak and Rogan (2010)	USA	Intellectual and Developmental Disabilities	Employment specialists	Workplace relations and organizational culture	Well-being	Intergroup contact theory
Sanclemente et al. (2020)	Spain	Applied Psychology	Employees with and without disabilities	Workplace relations and organizational culture	Sustainable employment	Affective events theory
Schaap et al. (2023)	Netherlands	Journal of Occupational Rehabilitation	EWD, supervisors	Leadership	Sustainable employment	
Schur et al. (2017)	USA	Journal of Occupational Rehabilitation	Employees with and without disabilities	Workplace relations and organizational culture; Leadership; Working conditions, health and safety	Well-being; Sustainable employment	Discrimination theories
Schur et al. (2009)	USA	Industrial Relations	Employees with and without disabilities	Workplace relations and organizational culture	Performance; Well-being; Sustainable employment	Stone and Colella’s model of factors affecting the treatment of EWD
Schur et al. (2020)	USA	Journal of Occupational Rehabilitation	Employees with and without disabilities	Accommodations	Performance	
Shuey and Jovic (2013)	Canada	Work and Occupations	EWD	Working conditions, health and safety	Well-being	Precariousness of nonstandard employment arrangements
Ulstein (2023)	Norway	Social Policy & Society	EWD	Practices general	Sustainable employment	
Uppal (2005)	Canada	Journal of Manpower	Employees with and without disabilities	Workplace relations and organizational culture; Working conditions, health and safety; Practices general	Well-being	
Villotti et al. (2017)	Australia, Canada,Italy	Community Mental Health Journal	EWD	Accommodations; Training and development EWD	Sustainable employment	
Zhu et al. (2019)	China	Human Resource Management	EWD	Workplace relations and organizational culture	Well-being	Self-efficacy theory

### Clustering Outcomes and Organizational Practices

Compared to the other outcomes clusters, the performance cluster covers the largest variety of outcomes. Some outcomes directly focus on performance: task performance [48], job performance [49, 50], creative performance [32], performance expectations [51], and performance evaluation [52]. Other outcomes we considered proxies of performance: hours worked [42, 43], and wages [40, 41, 53]. Two outcomes were of a more motivational nature in relation to performance: willingness to work hard [54], and thriving at work [55]. We decided to include willingness to work hard under performance as it was operationalized explicitly in terms of performance: “I am willing to work harder than I have to in order to help the company I work for succeed.” Thriving at work is being considered an antecedent of both performance and job satisfaction (part of our well-being outcome cluster; see below) [56]. Since it has been defined in terms of a ‘positive psychological state’ (see [56]) we included it under well-being. Performance ratings were measured in various ways, among others by asking employees or supervisors.

Most studies focusing on outcomes in the well-being cluster studied job [46, 47, 52, 54, 57, 58], work [59] or needs [48, 60] satisfaction. The other outcomes include positive experiences (resilience [52], self-esteem [50], work engagement [61], social integration [62], and thriving at work [55]) and negative experiences (emotional exhaustion [50], presenteeism [52] (1)) experiences of EWD. All studies of well-being involved EWD as respondents, sometimes together with employees without disabilities or supervisors when the studies also studied performance.

Studies in the sustainable employment cluster most frequently used job tenure [41–43, 57, 63] or, in longitudinal studies, employment status [34, 44, 45] as their outcome variable. Other outcomes included turnover intention [46, 54, 64] or its opposite: desire to stay [65]. Strictly speaking, job tenure, turnover intention and desire to stay are outcomes measuring sustainability of *jobs*, rather than of *employment.* Longitudinal studies looking at employment status come closer to measuring sustainability of employment. This has consequences for the interpretation of findings in terms of sustainable employment, as we will discuss below.

Some studies examined outcomes belonging to more than one outcome cluster. In some of these studies, relations between these outcomes were the subject of research as well.

Clustering the organizational practices resulted in eight clusters. The clusters workplace relations and organizational culture, accommodations and leadership appeared most frequently. The cluster *workplace relations and organizational culture* includes, among others, relations with colleagues, conditions of contact, fair treatment, discrimination, organizational inclusion, and team-learning climate. Studies in the *accommodations* cluster sometimes focus on job accommodations in general, sometimes on specific job accommodations such as schedule flexibility or telework. Studies that focused on *leadership* paid attention to transformational and moral leadership, leader-membership exchange and training of supervisors. The cluster *support of EWD* focused on aspects of the support provided to EWD, such as support provided by co-workers or job coaches and perceived organizational support. The cluster *working conditions, health and safety* includes studies analyzing the outcomes of job insecurity and threat of job loss, contract flexibility, and risk of accident or injury. The clusters *recruitment and selection* and *training and development* received limited attention. Limited attention to recruitment and selection may be considered logical, as this affects jobseekers with disabilities rather than EWD. Nevertheless, the study in our review that addressed recruitment and selection [57] showed that characteristics of the matching process may impact post-hiring outcomes. The final cluster, *practices in general*, refers to general characteristics of organizational practices, such as job demands, disability inclusive HR practices, and the extent of implemented disability policy.

As most of the studies focused on outcomes for EWD exclusively, it is difficult to answer the question whether the practices the studies examined were specifically targeted at EWD or at other groups of employees as well. Only some studies address this question explicitly. A study on telework [53] and two on accommodations [32, 34] examined outcomes of these practices for EWD and for people without disabilities. A number of studies examined practices specifically targeted at EWD [41, 42, 45] or at supervisors of EWD [44]. But even when practices are not disability-specific, studies of disparities between employees with and without disabilities showed that they may play out differently for different groups in organizations. A study by Schur and colleagues [46], among others, found that EWD scored lower on skill development and treatment by management than employees without disabilities. And Chandola and Rouxel’s study [34] of EWD and people without disabilities found that the largest predictor for having a work accommodation was having care responsibilities, not having an impairment.

When we compare the HPWP discussed earlier to our organizational practices clusters, recruitment and selection and training and development get attention, though limited in comparison with the other clusters. Some of our review articles did pay attention to compensation (wages), but as an outcome. The other HPWP (appraisal and performance management, employee involvement) were no topic of research in the articles. Organizational practices clusters not part of the HPWP are accommodations; workplace relations and organizational culture; working conditions, health and safety; and support for EWD. The final organizational practices cluster, leadership, is often not considered as an HR practice. HR scholars’ plea to integrate the study of HR and leadership [66] is already visible in studies of organizational practices and their outcomes for EWD.

### Synthesis of Findings

Table 2 provides an overview of the significant positive, significant negative, and non-significant relations between practices and outcomes as reported in the articles. We will discuss the findings for each of the outcome clusters separately.

**Table 2:** Findings concerning relationships between organizational practices and outcomes clusters

Organizational practices clusters	Outcomes clusters		
	Performance	Well-being	Sustainable employment
*Significant positive relations*			
Recruitment and selection		Eissenstat et al. (2022)	
Workplace relations and organizational culture	Banks et al. (2001) Schur et al. (2009) de Carvalho-Freitas et al. (2017)	Gray et al. (2014) Schur et al. (2009) Schur et al. (2017) Novak and Rogan (2010) Zhu et al. (2019)	Chordiya (2020) Sanclemente et al. (2022) Schur et al. (2009) Schur et al. (2017)
Accommodations	Chow et al. (2014) de Carvalho-Freitas et al. (2023) Man et al. (2020)	Eissenstat et al. (2022) Gray et al. (2014)	Chandola and Rouxel (2021) Chow et al. (2014)
Support of EWD	Coll and Mignonac (2023) Mank et al. (2000)	Coll and Mignonac (2023) Gray et al. (2014)	Mank et al. (2000)
Training and development EWD			Villotti et al. (2017)
Leadership	Kensbock and Boehm (2016) Lyubykh et al. (2020)	Kensbock and Boehm (2016) Luu (2018) Lyubykh et al. (2020) Schur et al. (2017)	Schaap et al. (2023) Schur et al. (2017)
Working conditions, health and safety			
Practices general		Flores et al. (2021) Luu (2018)	Ulstein (2023)
*Significant negative relations*			
Recruitment and selection			
Workplace relations and organizational culture		Eissenstat et al. (2022) Uppal (2005)	Chordiya (2020) Eissenstat et al. (2022)
Accommodations			
Support of EWD	Mank et al. (2000)		Mank et al. (2000)
Training and development EWD			
Leadership			
Working conditions, health and safety		Schur et al. (2017) Shuey and Jovic (2013) Uppal (2005)	Schur et al. (2017)
Practices general		Flores et al. (2021) Uppal (2005)	
*No significant relation*			
Recruitment and selection		Eissenstat et al. (2022)	
Workplace relations and organizational culture			Chordiya (2020)
Accommodations	Schur et al. (2020)		
Support of EWD	Farris and Stancliffe (2001)		Farris and Stancliffe (2001)
Training and development EWD			
Leadership			
Working conditions, health and safety		Schur et al. (2017)	
Practices general			

#### Performance

The twelve studies in our review examining performance addressed four clusters of organizational practices. Concerning *workplace relations and organizational culture*, Banks and colleagues [40] examined interactions with colleagues and found that the amount of social interaction related positively to the performance of EWD. De Carvalho-Freitas and Stathi [51] conducted an experimental study testing a specific intervention that could be used as an organizational practice: the imagined contact intervention, which aims to influence attitudes. The study demonstrated that the intervention resulted in more positive expectations of work-related outcomes of EWD. Schur and colleagues [54] examined the role of organizational culture more broadly and found that perceived fair company treatment and company responsiveness to employee concerns reduced the gap in willingness to work hard between employees with and without disabilities.

Two studies [32, 43] found a positive impact of *accommodations* on EWD’s performance. De Carvalho-Freitas and colleagues [49] found a similarly positive relationship for adaptations of working conditions and practices. Schur and colleagues examined a specific work accommodation: telework [53]. They found that EWD experience similar wage gaps with their non-disabled colleagues in on-site and home-based work, concluding that telework does not reduce this gap.

The relationship between *support for EWD* and performance was analyzed in three studies. Coll and Mignonac [48] found that perceived organizational support related positively to EWDs’ performance. Mank and colleagues [41] found that the negative relationship between the hours of support provided by co-workers and EWD’s wages can be reduced by providing co-workers with training. Farris and Stancliffe [42] compared support provided by job coaches and by co-workers and found no difference in the relation between support and EWD’s performance when comparing both types of support.

Two studies looked at *leadership*. Kensbock and Boehm [50] found positive relations between transformational leadership and EWD’s performance. Lyubykh and colleagues [52] analyzed the role of leader-member exchange, i.e., the quality of employee-supervisor relationships, and found a positive relationship with supervisors’ evaluation of EWD’s performance.

#### Well-Being

Thirteen studies analyzed relationships between organizational practices and well-being. Eissenstat and colleagues [57] examined the effects of characteristics of *recruitment and selection* processes on the job satisfaction of EWD. The study found that education-level match and aptitude match are positively related to job satisfaction. No significant relation was found between job-skill match and job satisfaction. Several studies examined practices in the *workplace relations and organizational culture* cluster. The studies of Schur and colleagues and of Gray and colleagues found that co-worker communication [59] and co-worker relations [46] contributed to job satisfaction. Zhu and colleagues [55] found that workplace inclusion and team climate reduced the negative effect of disability on self-efficacy, which has a positive effect on thriving at work. Schur and colleagues [54] found a positive relationship between aspects of organizational culture (i.e., fair company treatment and responsiveness to employees’ needs) and job satisfaction. The study of Novak and Rogan [62] found positive relationships between conditions of contact and workplace culture on the one hand, and well-being (social integration) of EWD on the other. Workplace relations and organizational culture can also affect job satisfaction negatively: through discrimination [47, 57], harassment and poor interpersonal relationships [47].

Concerning *accommodations*, studies found positive relationships between job satisfaction and worksite accessibility [59], work tasks [59], and accessible work facilities [57]. As far as the *support of EWD* cluster is concerned, perceived organizational support [48] and worksite support [59] were found to be positively related to job satisfaction. The same goes for *leadership*, specifically leader-member exchange [52], treatment by management [46], and moral leadership [61]. Characteristics of leadership can also reduce negative experiences of EWD. The quality of leader-member exchange reduced levels of presenteeism (see note 1) [52], and transformational leadership can prevent emotional exhaustion by contributing to EWD’s organization-based self-esteem [50]. Studies focusing on the cluster *working conditions, health and safety* have mainly highlighted practices that were found to jeopardize job satisfaction: flexible, non-standard contracts [46, 60] and the threat of job loss and risk of accident or injury [47]. Schur and colleagues [46] did not find a significant (direct) relationship between job security and job satisfaction.

Finally, three studies looked at more *general practices*. Two studies found that (too) high job demands are negatively related to job satisfaction [47, 58], whereas job resources are positively related [58]. The third study [61] found a positive relationship between disability inclusive human resource practices and work engagement, moderated by organizational identification.

#### Sustainable Employment

Twelve studies investigated outcomes in the sustainable employment cluster. As turnover intention may be interpreted as a negative indicator of sustainable employment, studies finding a negative relation between an organizational practice and turnover intention were entered under ‘significant positive relations’ in Table 2.

Five studies looked at *workplace relations and organizational culture*. Chordiya [64] studied several elements of inclusive organizational culture and found that fairness was positively related to sustainable employment. A negative relationship was found for supportiveness and empowering, whereas no significant results were obtained for openness to diversity and cooperative management. Schur et al. [54] found positive relationships with sustainable employment for fairness (confirming the former study) and responsiveness to employees’ concerns. Sanclemente and colleagues [65] found that organizational socialization tactics were positively related to sustainable employment. Co-worker relations were positively related to sustainable employment in a study by Schur et al. [46], whereas EWD’s experiences of discrimination had the opposite effect [57].

Several studies found positive relations between sustainable employment and *accommodations* more generally [34, 43] or specific accommodations: accessible work facilities [57] and schedule flexibility [63]. Two studies focused on *support for EWD*. Mank [41] found that training of co-workers could buffer the negative effect of hours of co-worker support on sustainable employment. Farris and Stancliffe [42] found that providing support by job coaches or co-workers did not matter in terms of the effect of support on sustainable employment.

The only study in the *training and development* cluster [63] found that training of EWD was positively related to job tenure. *Leadership* in relation to sustainable employment received attention in two studies. Schur and colleagues [46] found a positive relationship between treatment by management and sustainable employment. Schaap et al. [44] found indications of a positive contribution of supervisor training to sustainable employment. In the *practices general* cluster, Ulstein’s study [45] found a positive relationship between the extent of implemented disability policy and sustainable employment of EWD.

The interpretation of outcomes in terms of sustainable employment requires some side notes. First, since most studies—as mentioned before—examine sustainability of jobs, negative outcomes in terms of job sustainability do not necessarily imply negative outcomes in terms of sustainability of employment. EWD leaving their jobs may do so because they found better jobs elsewhere. Secondly, from the perspective of EWD job sustainability may not necessarily be positive. It may also imply lack of alternative employment options. The studies in our review did not provide evidence of this, but only one study [57] looked at the relationship between well-being (job satisfaction) and sustainable employment (job tenure)—the study found a positive relationship.

### Theoretical Perspectives

Finally, we examine the theoretical perspectives the studies used in understanding the relations between organizational practices and outcomes. We focus on articles in which an explicit theoretical perspective was guiding the research questions or hypotheses.

Many of the theoretical perspectives have in common that they focus on the interaction between individuals and their environment. In the studies the individuals are employees with disabilities; their environment consists of the organizations in which they work, characteristics of their work, supervisors, and colleagues. Thus, the theoretical focus in the studies reflects the interactional definition of disability.

Two studies explicitly used the World Health Organization’s International Classification of Functioning, Disability, and Health (ICF), that laid the foundation for this interactional concept of disability. Both studies hypothesized that work accommodations will enhance the person-environment fit between EWD and their workplace, which will improve EWD’s performance [43] and sustainable employment [34, 43]. Eissenstat and colleagues [57] and Gray et al. [59] used the theory of work adjustment in hypothesizing that the fit between person and environment is reflected in employees’ job satisfaction, which will contribute to their job retention [57]. Adopting a more psychological perspective, two studies used Bandura’s self-efficacy theory and hypothesized that accommodations [32] and workplace inclusion and leadership [55] will have a positive impact on EWD’s performance [32] and well-being [55] through their positive impact on EWD’s self-efficacy. Focusing on creative performance specifically, Man’s study also drew upon the componential model of individual creativity [32], arguing that work environments affect the motivational and skill-related components that contribute to individual creativity. In as far as work accommodations facilitate these components, they contribute to performance. Another psychological approach is used in two studies inspired by the Job Demands-Resources theory, which explains how the work environment impacts employee well-being and performance. The studies hypothesized that job resources contribute to work engagement [61] and job satisfaction [58]. In addition, Flores and colleagues [58] hypothesized that high job demands relate to job satisfaction negatively. Luu [61] also used social identity theory to explain why by investing in resources, organizations may enhance EWD’s work engagement through strengthening their organizational identification. Yet another psychological approach was applied in the study of Coll and Mignonac [48] who took Ryan and Deci’s basic psychological needs theory as their starting point. This theory argues that individuals have a limited set of basic psychological needs, the satisfaction of which is essential for their well-being. Coll and Mignonac expected that perceived organizational support will increase EWD’s basic need satisfaction and in doing so, contribute to their performance.

Four studies used a social interaction theoretical perspective. Lyubykh et al. [52] drew upon social exchange theory, which focuses on the costs and benefits of actors involved in interaction. The authors argued that if employees experience favorable treatment by leaders, they will reciprocate this treatment. Consequently, high quality leader-member exchange is hypothesized to be related positively to employee well-being and performance. The studies applied intergroup contact theory that argues that positive and frequent contacts among people will improve judgments and reduce prejudice. Based on this theory, Carvalho-Freitas and Stathi [51] developed an imagined contact intervention, hypothesizing that it will result in more positive expectations of EWD’s performance. Novak and Rogan [62] expanded on contact theory’s tenet that good contact conditions contribute to attitudes toward EWD by hypothesizing that this in turn will strengthen EWD’s social integration. Sanclemente and colleagues [65] used the affective events theory. According to this theory, the experience of affective states at work depends on the work situations experienced by employees throughout their working life. Applied to their research, the authors hypothesized that organizational socialization tactics will produce affective emotions in EWD in terms of feeling included that strengthen their desire to stay in the organization.

Two studies made use of discrimination theories to explain disparities between employees with and without disabilities in employee outcomes such as job satisfaction and turnover intention [46, 64]. Both studies argued that taste-based and statistical discrimination theories are helpful in understanding the treatment of EWD in organizations. Schur and colleagues [54] used Stone and Colella’s model of factors affecting the treatment of EWD. The study focused on corporate culture and hypothesized that bureaucratic organizations with rigid value systems will perform worse in terms of EWD’s employee outcomes than flexible organizations that value diversity, cooperation, and personal attention to employee needs. Finally, Shuey and Jovic [60] used a sociological theoretical perspective, namely non-standard employment arrangements and their precariousness. The authors hypothesized that if the provision of disability work accommodations reflects the unequal distribution of other labor market protections, it will result in unmet accommodation needs of EWD in non-standard employment arrangements.

## Conclusion and Discussion

In this section, we will conclude and discuss our results related to each of the three steps of our analysis. Studies of organizational practices and their outcomes for EWD focused on three of the eight organizational practices clusters we distinguished in particular: workplace relations and organizational culture; accommodations; and leadership. Each of these clusters has been studied in relation to all the outcomes we distinguished. Comparing these clusters to the HPWP often studied in HRM studies, we found that the overlap was modest. The HPWP of recruitment and selection and of training and development received limited attention, while the organizational practices receiving most attention in the articles in our review (accommodations, workplace relations and organizational culture, and leadership) are generally not regarded as HPWP. This raises some interesting issues for future research. First, the limited attention to the impact of HPWP on EWD does not tell us anything about the importance of these HPWP for EWD. Therefore, more systematic research into the outcomes of HPWP for EWD is recommended (also see [19]). For example, Hoque and colleagues [35] found that although HPWP may negatively impact the hiring of people with disabilities, EWD working in organizations with more HPWP have higher work-related well-being compared to EWD in organizations with few HPWP. This indicates that EWD may, though not necessarily do, benefit from HPWP. Thus, more insight into the positive and negative outcomes of HPWP for EWD can support HR decision-making in organizations. At the same time, we recommend studying whether organizational practices that have positive outcomes for EWD also do so for wider populations of employees. If this turns out to be the case—and we consider that very likely—it may reduce barriers for employers to implement these practices. For employers will be more likely to implement HR practices as these produce positive outcomes among larger groups of employees. In addition, studies of disparities between EWD and people without disabilities made clear that the accessibility of ‘general’ practices for various groups of employees needs attention too.

A limitation of our review is that we summarized the findings in terms of significant relationships that were found in the studies, without consideration of effects sizes, covariates et cetera. With that limitation in mind, the findings of the studies support the proposition that organizational practices matter in terms of EWD’s performance, well-being, and sustainable employment. Furthermore, the organizational practices that received most attention revealed positive relationships with each of the three outcomes this review examined. At the same time, organizational practices may also have a disabling effect. We found these effects specifically in aspects of the workplace relations and organizational culture and the working conditions, health & safety clusters. ‘Hidden’ negative outcomes (lack of alternative options) may be present in positive outcomes in terms of sustainable employment; the studies in our review did not examine this issue in depth. For researchers, this means that future research into the outcomes of organizational practices for EWD may be fruitful. For employers, it means that investing in enabling practices and discouraging disabling practices pays off in terms of organizational success: EWD that perform well, are satisfied with their jobs, and desire to stay in the organizations where they work add more value to the organization.

Despite these promising findings, the available evidence is still limited and fragmented. Our clustering of practices and outcomes was an attempt to abstract from the variety of practices and outcomes that have been studied, but that does not mean that variety is not relevant. Furthermore, as yet we are unable to determine the impact that contextual factors such as type or size of organizations might have on the relationships between organizational practices and their outcomes. Therefore, future research should not only be broader, including more organizational practices and groups of employees. It should also be deeper, examining specific practice-outcome combinations, preferably in various contexts. In addition, we recommend future researchers to examine the relationship between job and employment sustainability, as well as EWD’s perspective on job sustainability as a positive or negative experience.

Finally, on a more positive note, the empirical findings reported in the articles were in most cases in line with what was expected based on the theories used in the studies. Together with the variety of theories that were used, this strengthens the validity of these findings. Theories used in the studies have in common that they focus on environmental factors and their impact on individuals, thus relating directly to the interactional concept of disability. The overarching tenet is that environments may provide—or fail to provide—individuals with the opportunities, resources, meaningful experiences or affective states that they need to perform, feel well, and able and willing to retain their jobs. The environment is studied at different levels: the micro-level of interactions between employees and their colleagues and supervisors; the meso-level of organizations and how they facilitate or hinder positive employee outcomes; and the macro-level of societal inequalities in the distribution of opportunities and resources. Interestingly, most theories do not specifically focus on people with disabilities but address employees (or individuals) in general. This strengthens our assumption that organizational practices that turn out to be beneficial for EWD may also be beneficial for other (groups of) employees, making it more attractive for employers to implement them.

The variety of theories made it difficult to identify clear links between the theoretical perspectives and the outcomes being studied. Whether these links could be expected to exist in the first place may be questioned given the fact that the outcomes themselves are related. Studies have pointed, for example, at relationships between job satisfaction on the one hand and performance [67] and turnover intention [68] on the other. Some articles in our review found similar relationships [48, 50, 57].

More systematic research into the outcomes of organizational practices for EWD will increase our understanding of what practices work for whom under what circumstances. It will also support professionals in the areas of rehabilitation, HRM, and reintegration in developing and implementing evidence-based interventions to make workplaces more responsive to EWD’s needs.

## Notes

1. Presenteeism is defined by the authors as being present at work despite an illness or injury, and is therefore considered a negative experience of EWD.

## Supplementary Information

Below is the link to the electronic supplementary material.

Supplementary file1 (DOCX 40 KB)

Supplementary file2 (DOCX 39 KB)

Supplementary file3 (DOCX 20 KB)

## Data Availability

No datasets were generated or analyzed during the current study.
