# Supplementary material for: Organizational Practices and Their Outcomes for Employees with Disabilities: A Review and Synthesis of Quantitative Studies
Source: J Occup Rehabil. 2025 Mar 12;36(1):117–30. doi: 10.1007/s10926-025-10283-6 (PMC12906511; doi:10.1007/s10926-025-10283-6)
Supplement: Supplementary file 1 — Supplementary file1 (DOCX 40 KB) [file 10926_2025_10283_MOESM1_ESM.docx]

ORGANIZATIONAL PRACTICES AND THEIR OUTCOMES FOR EMPLOYEES WITH DISABILITIES. A REVIEW AND SYNTHESIS OF QUANTITATIVE STUDIES

Journal of Occupational Rehabilitation

Rik van Berkel, Eric Breit

[r.vanberkel@uu.nl](mailto:r.vanberkel@uu.nl)

**Appendix 1. Scoping review flow chart**

Literature search PsychINFO, Web of Science, Sociological Abstracts and Sociological Index*

kkkkkkkkk

N = 146

Full text screening, excluding: not related to organizational inclusion practices and PWD, focus on RTW

N = 871

Excluding articles in journals with no impact factor

N = 977

Screening titles/abstracts, excluding: not related to organizational inclusion practices, not related to PWD, focus on RTW

N = 10,535

N = 12,538

Removal of duplicates

*Full search string:

**Target group:** disab* OR impair* OR handicap*

AND

**Workplace practice:** job accommodation* OR job adjustment* OR job modification* OR workplace accommodation* OR workplace adjustment* OR workplace modification* OR workplace train* OR job coach* OR intervention* OR HRM polic* OR HRM practice* OR support* OR vocation* OR rehabilitation*

AND

**Employment outcome:** employ* OR work* OR job OR vocation* OR occupation* OR labo?r-market participation OR competitive employ* OR supported employ* OR incl* OR diversity OR integration

AND

**NOT:** child* OR ag?ing OR student* OR gerontology* OR pregnan*
