# Supplementary material for: Organizational Practices and Their Outcomes for Employees with Disabilities: A Review and Synthesis of Quantitative Studies
Source: J Occup Rehabil. 2025 Mar 12;36(1):117–30. doi: 10.1007/s10926-025-10283-6 (PMC12906511; doi:10.1007/s10926-025-10283-6)
Supplement: Supplementary file 2 — Supplementary file2 (DOCX 39 KB) [file 10926_2025_10283_MOESM2_ESM.docx]

ORGANIZATIONAL PRACTICES AND THEIR OUTCOMES FOR EMPLOYEES WITH DISABILITIES. A REVIEW AND SYNTHESIS OF QUANTITATIVE STUDIES

Journal of Occupational Rehabilitation

Rik van Berkel, Eric Breit

[r.vanberkel@uu.nl](mailto:r.vanberkel@uu.nl)

**Appendix 2. Review flow chart**

Scoping review (see appendix 1)

kkkkkkkkk

N = 30

Including articles using quantitative methods; excluding qualitative studies

N = 49

Exclusion of articles not addressing the following outcomes: performance/productivity; well-being; sustainable employment

N = 59

N = 146

Articles focusing on organizational practices for EWD and their outcomes

Quality assessment; Excluding descriptive articles

N = 27
