# Supplementary material for: Organizational Practices and Their Outcomes for Employees with Disabilities: A Review and Synthesis of Quantitative Studies
Source: J Occup Rehabil. 2025 Mar 12;36(1):117–30. doi: 10.1007/s10926-025-10283-6 (PMC12906511; doi:10.1007/s10926-025-10283-6)
Supplement: Supplementary file 3 — Supplementary file3 (DOCX 20 KB) [file 10926_2025_10283_MOESM3_ESM.docx]

ORGANIZATIONAL PRACTICES AND THEIR OUTCOMES FOR EMPLOYEES WITH DISABILITIES. A REVIEW AND SYNTHESIS OF QUANTITATIVE STUDIES

Journal of Occupational Rehabilitation

Rik van Berkel, Eric Breit

[r.vanberkel@uu.nl](mailto:r.vanberkel@uu.nl)

**Appendix 3. Journal rankings (SJR 2023)**

| Article | Journal | Subject area and category | SJR ranking: Quartile 2023 |
| --- | --- | --- | --- |
| Banks et al., 2001 | Psychiatric Rehabilitation Journal | Medicine -> Rehabilitation | Q1 |
| Chandola & Rouxel, 2021 | Social Science & Medicine | Social Science -> Health | Q1 |
| Chordiya, 2020 | Review of Public Personnel Administration | BMA -> Organizational Behaviour (OB) and HRM | Q1 |
| Chow et al., 2014 | Psychiatric Services | Medicine -> Psychiatry and Mental Health | Q1 |
| Coll & Mignonac, 2023 | International Journal of Human Resource Management | BMA -> OB and HRM | Q1 |
| de Carvalho-Freitas et al., 2023 | Applied Psychology - an International Review | Psychology -> Applied psychology | Q1 |
| de Carvalho‐Freitas & Stathi, 2017 | Journal of Applied Social Psychology | Psychology -> Social Psychology | Q2 |
| Eissenstatt et al., 2022 | Rehabilitation Counseling Bulletin | Medicine -> Rehabilitation | Q2 |
| Farris & Stancliffe, 2001 | Journal of Intellectual & Developmental Disability | Medicine -> miscellaneous | Q2 |
| Flores et al., 2021 | International Journal of Environmental Research and Public Health | Medicine -> Public Health, Environmental and Occupational Health | Q2 |
| Gray et al., 2014 | Work | Medicine -> Rehabilitation | Q2 |
| Kensbock & Boehm, 2016 | International Journal of Human Resource Management | BMA -> OB and HRM | Q1 |
| Luu, 2018 | Employee Relations | BMA -> OB and HRM | Q2 |
| Lyubykh et al., 2020 | Journal of Occupational Rehabilitation | Medicine -> Rehabilitation | Q1 |
| Man et al., 2020 | Frontiers in Psychology | Psychology -> miscellaneous | Q2 |
| Mank et al., 2000 | Mental Retardation* | Medicine -> Psychiatry and Mental Health | Q2 |
| Novak & Rogan, 2010 | Intellectual and Developmental Disabilities | Medicine -> Psychiatry and Mental Health | Q2 |
| Sanclemente, 2022 | Applied Psychology - an International Review | Psychology -> Applied psychology | Q1 |
| Schaap et al., 2023 | Journal of Occupational Rehabilitation | Medicine -> Rehabilitation | Q1 |
| Schur et al., 2017 | Journal of Occupational Rehabilitation | Medicine -> Rehabilitation | Q1 |
| Schur et al., 2009 | Industrial Relations | BMA -> OB and HRM | Q1 |
| Schur et al., 2020 | Journal of Occupational Rehabilitation | Medicine -> Rehabilitation | Q1 |
| Shuey & Jovic, 2013 | Work & Occupations | BMA -> OB and HRM | Q1 |
| Ulstein, 2023 | Social Policy & Society | Social Science -> Sociology and Political Science | Q2 |
| Uppal, 2005 | International Journal of Manpower | BMA -> OB and HRM | Q1 |
| Villotti et al., 2017 | Community Mental Health Journal | Medicine -> Public Health, Environmental and Occupational Health | Q1 |
| Zhu et al., 2019 | Human Resource Management | BMA -> OB and HRM | Q1 |

* currently Intellectual and Developmental Disabilities
